# Cytotoxic activity of phenolic compounds in Bairui Granules obtained from the Chinese medicinal plant *Thesium chinense*

**DOI:** 10.3389/fchem.2024.1506792

**Published:** 2024-12-02

**Authors:** Shaobin Zhang, Hong Chen, Juan Hua, Shihong Luo

**Affiliations:** Engineering Research Center of Protection and Utilization of Plant Resources, College of Bioscience and Biotechnology, Shenyang Agricultural University, Shenyang, Liaoning, China

**Keywords:** *Thesium chinense* Turcz., phenolic compounds, Bairui granules, active substances, cytotoxic activity

## Abstract

The Chinese medicinal plant *Thesium chinense* Turcz. is the only plant used in the manufacture of Bairui Granules. However, to date, there has been very little research into the cytotoxic activity of active substances derived from Bairui Granules. Using chemical separation and spectroscopic methods, phenolic compounds 1–5 were identified as methyl-*p*-hydroxycinnamate, vanillin, kaempferol, isorhamnetin-3-*O*-glucoside, and astragalin, respectively. UPLC-MS/MS analyses revealed that compounds 1–5 were present at concentrations of 0.006 ± 0.002, 1.63 ± 0.87, 3.65 ± 0.83, 26.97 ± 11.41, and 27.67 ± 2.91 *μ*g/g, respectively in Bairui Granules. Compounds 1, 2, and 4 were detected here for the first time in Bairui Granules. Using co-culture experiments, isorhamnetin-3-*O*-glucoside (4) was found to be beneficial to the proliferation Chinese hamster ovary (CHO) cells (6.46% ± 0.86% to 38.45% ± 9.04%), natural killer cells from human umbilical cord blood (UCB NK cells) (25.68% ± 0.02% to 70.81% ± 0.26%), and mesenchymal stem cells from human umbilical cord blood (UCB MSC cells) (1.66% ± 0.05% to 27.64% ± 0.51%) when the concentration was similar to that found in Bairui granules. Moreover, vanillin (2) was conducive to UCB NK cells proliferation (28.21% ± 0.44%) at a concentration of 64 *μ*g/mL, while maintaining cell viability. UCB NK cell proliferation was promoted at rates of 41.03% ± 0.48% to 67.22% ± 0.68% when astragalin (5) was present at low concentrations (8 and 16 *μ*g/mL). Methyl-*p*-hydroxycinnamate (1) and vanillin (2) at different concentrations both had an inhibitory effect on the proliferation of natural killer cells from human peripheral blood (PB NK cells), but the inhibitory concentration ranges of these compounds were not equivalent to the concentration ranges of the compounds in Bairui Granules. These results provide a foundation for the safe use of *T. chinense* preparations.

## Graphical Abstract

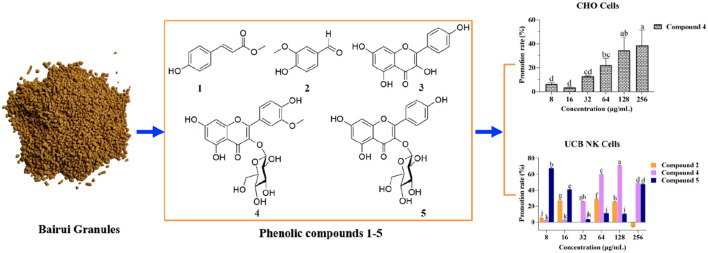

## 1 Introduction

*Thesium chinense* Turcz. Is a perennial herbaceous plant belonging to the Satalaceae family, and is widely distributed in eastern Asia (Shao et al., 2019). In China, *Thesium chinense* is used as a traditional Chinese medicine with many years of history, and described recorded in several Chinese ethnomedical monographs (Li G. H. et al., 2021). As early as the Song Dynasty, the Ben Cao Tu Jing described the usage and required dosage of *T. chinense* for clearing heat and detoxifying, tonifying the kidneys, and as an astringent essence (Liu et al., 2023). In 1950, other uses of *T. chinense*, including the treatment of head sores and cervical lymphadenitis, were recorded in the Guo Yao Ti Yao (Li G. H. et al., 2021). The decoction of the whole plant is used to treat mastopathy, headache resulting from pneumonia, stomach pain, and tuberculosis of cervical lymph nodes in Anhui and Fujian Provinces (Li G. H. et al., 2021; Parveen et al., 2007). Furthermore, *T. chinense* has also been made into modern pharmaceutical preparations, including Bairui Granules and Bairui Tablets, which are mainly used to treat tracheitis, rhinitis and colds. The Bairui Granules sales more than 400 million yuan in the last year. *T. chinense* has therefore played an important role as a Chinese herbal medicine at least since the Song Dynasty.

Because *T. chinense* has such widespread traditional uses, the active chemical substances in the plant have received widespread attention. *T. chinense* has been found to contain abundant secondary metabolites, including flavonoids, organic acids, alkaloids, and terpenoids (Liu et al., 2018; Lombard et al., 2020). The various biological activities of these chemical substances have been studied in *in vitro* and *in vivo* experiments (Li G. H. et al., 2021). The flavonoids are now considered to be the main functional substances in *T. chinense* (Cao et al., 2021), and total flavonoids and kaempferol are now taken to be the quality control standards for traditional medicinal preparations of *T. chinense* in China. These flavonoids not only exhibit anti-inflammatory and antioxidant activities, and inhibit the proliferation of cancer cells, but have also been found to play a certain role in protecting the liver and nerves (Khan et al., 2020; Soromou et al., 2012; Zhen et al., 2017). The phenolic compounds isolated from *T. chinense* also exhibited certain anti-inflammatory effects (Liu et al., 2023). The positive therapeutic effects of *T. chinense* are therefore likely to be closely related to the chemical substances it contains. However, there is little research on the active substances present in Bairui Granules, which are prepared with *T. chinense* as their sole herbal ingredient.

The human body contains many types of immune cells and stem cells, including natural killer cells (NK cells) and mesenchymal stem cells (MSC cells) (Musina et al., 2005; Strowig et al., 2010). NK cells have the ability to clear leukemia and tumor cells, and MSC cells have strong hematopoietic support function and are able to differentiate into various different types of human cells as required (Steward and Kelly, 2014; Vyas et al., 2023). Chinese hamster ovary cells (CHO cells) are the most widely applied mammalian recombinant protein cell line in immunology (Róisín et al., 2020). However, it is not yet known whether the chemical substances present in *T. chinense* have an impact on human immune cells or stem cells. In this study, we focused on flavonoids and phenolic compounds present in Bairui Granules and investigated whether these have an impact on mammalian expression vectors, immune cells, and stem cells, further providing reference for the correct and safe dosage in preparations of *T. chinense*.

## 2 Materials and methods

### 2.1 Plant materials and test cell lines

Whole dried *T. chinense* plants were purchased from the Hebei Baoding Anguo Medicinal Material Wholesale Market, and were identified by Professor Bo Qu. Chinese hamster ovary cells (CHO cells), natural killer cells from human umbilical cord blood (UCB NK cells), natural killer cells from human peripheral blood (PB NK cells), and mesenchymal stem cells from human umbilical cord blood (UCB MSC cells) were provided by the Cell Preparation Center of Qinhuangdao Weiming Health City Development Co., Ltd. CHO cells were cultured in RPMI-1640 medium (Hyclone) containing 10% fetal bovine serum (Gibco). NK cells and MSC cells were cultured with the corresponding cell culture kits. The NK and MSC cell culture kits were purchased from the Beijing Tongli Haoyuan Biotechnology Co., Ltd. FITC-conjugated anti-human CD3 antibody and APC-conjugated anti-human CD56 antibody were purchased from Meitianni Biotechnology Co., Ltd.

### 2.2 Isolation and identification of phenolic compounds from *Thesium chinense*

The entire dried *T. chinense* plants (15.0 Kg) were subjected to a methanol extraction. The resulting extract was then concentrated under a vacuum and was treated with an equal volume of ethyl acetate (1:1, v/v). The crude ethyl acetate fraction (204.0 g) was purified on a silica gel column using eluents (dichloromethane/methanol, 1:0−0:1), and resulting in five fractions (Fr.-1−Fr.-5). Fr.-2 (29.0 g) was passed through an MCI gel column chromatography column (methanol/water, 70:30−100:0) to get four parts (Fr.-2.1−Fr.-2.4). Fr-2.1 (19.4 g) was separated on a silica gel column with petroleum ether/acetone elution (15:1, v/v) to obtain six fractions (Fr.-2.1.1−Fr.-2.1.6). Fr.-2.1.5 (4.5 g) and Fr.-2.1.6 (6.5 g) were passed separately through a Sephadex LH-20 column to get four parts (Fr.-2.1.5.1−Fr.-2.1.5.4) and five parts (Fr.-2.1.6.1−Fr.-2.1.6.5), respectively. Fr.-2.1.5.2 (320.0 mg) was purified using semi-preparative HPLC with DAD detector (methanol/water, 45:55) to yield compound 1 (50.7 mg). Fr.-2.1.5.3 (452.2 mg) was purified using semi-preparative HPLC with DAD detector (methanol/water, 25:75) to yield compound 2 (90.4 mg). Fr.-2.1.5.4 (783.0 mg) was purified using semi-preparative HPLC with DAD detector (methanol/0.2% acid water, 58:42) to yield compound 3 (110.4 mg), and Fr.-2.1.6.3 (2.4 g) was purified using semi-preparative HPLC with DAD detector (methanol/0.2% acid water, 41:59) to yield compounds 4 (156.0 mg) and 5 (175.3 mg).

### 2.3 Qualitative and quantitative analysis of compounds one to five in Bairui Granules

Samples of Bairui Granules (Anhui Jiuhua Huayuan Pharmaceutical Co., Ltd.) were prepared for detection of the compounds. About 0.2 g sample was subjected three times in succession to a methanol extraction in an ultrasonic bath, each time for 45 min. The obtained methanol extract was then concentrated under vacuum until dry. Then, the dissolved liquid (500 *μ*L chromatographic methanol) was filtered through 0.22 *μ*m filter, and the prepared samples were finally analyzed on the UPLC-MS/MS (Shimadzu LCMS-8050). Briefly, 10 *μ*L samples were injected into Shim-pack GIST C_18_ column (2 *μ*m, 100 × 2.1 mm, 0.2 mL/min), and the column temperature was maintained at 40 °C. A (0.1% acid water) and B (acetonitrile) were selected as mobile phases, and the elution procedure was as follows: 0–12 min, from 5% B to 95% B; 12–13 min, 95% B; 13–14 min, from 95% B to 5% B; 14–17 min, 5% B. The ESI procedure was as follows: dry gas flow rate 10 L/min, heating block temperature 450 °C; interface temperature 300 °C; heating gas flow rate 10 L/min; atomizing gas flow rate 3 L/min; (Yang et al., 2022). Compounds one to five were monitored using multiple reaction monitoring (MRM) mode, and their related parameters are given in Supplementary Table S1. The calibration curves for compounds one to five are shown in Supplementary Table S2.

### 2.4 *In vitro* cytotoxicity assay

The compounds to be assayed were dissolved in DMSO (0.5% of the total volume). Four cell suspensions (CHO cells, UCB NK cells, PB NK cells, and UCB MSC cells) were prepared in advance. Each of the compounds was diluted to the final concentrations: 8, 16, 32, 64, 128, and 256 *μ*g/mL, and were added to a 24-well plate, in which compound 4 prepared for CHO cells, compounds 2, 4, and 5 prepared for UCB NK cells, compounds 1, 2, and 4 prepared for PB NK and UCB MSC cells. Each well included cell suspension, cell culture medium and compound solution, at the ratio of 100:99:1. 500 *μ*L of cell culture medium/DMSO (99:1, v/v) was added to 500 *μ*L of cell suspension as a control (Li et al., 2022). The number of cells in each well was approximately 1.0E+06, and each treatment included three biological replicates. The co-cultured cell suspensions were then cultured in a carbon dioxide incubator (5% CO_2_, 37°C, saturated humidity) for 48 h. After the cultivation step was complete, 10 *μ*L of co-cultured cell suspension was added to 0.4% trypan blue (10 *μ*L) and a uniform mixture was prepared. Subsequently, 10 *μ*L of this mixture was then injected into each cavity of the slide and maintained for 30 s. Finally, the cell morphology was observed using an inverted microscope (Nikon) and cells were counted using a cell counter (Thermo).

### 2.5 Detection of PB NK cell surface markers

PB NK cells were cultured for 48 h according to the method described above. The co-cultured cell suspension (about 1.0E+06) was moved to a EP tube (1.5 mL). The samples were centrifuged at 1,500 rpm for 3 min, an then the supernatant was removed and discarded. The remaining cells were re-suspended using 1,000 *μ*L of phase buffer saline (PBS) and then centrifuged at 1,500 rpm for 3 min. The supernatant was then removed and discarded. This process was repeated twice. Finally, the obtained cells were re-suspended in 1,000 *μ*L of PBS to obtain a new cell suspension. 100 *μ*L of the new cell suspension was injected into each of three EP tubes, with one tube containing 5 *μ*L of FITC-conjugated anti-human CD3 antibody, one containing 5 *μ*L of APC-conjugated anti-human CD56 antibody, and with one tube serving as a blank control (5 *μ*L PBS). Evenly mixed samples were incubated at 4 °C in the dark for 10 min. After incubation, each tube of cells was re-suspended using the above method, resulting in a new cell suspension with a final volume of 500 *μ*L. Finally, the samples were analyzed using flow cytometry (MiltenyiBiotecB. V. and Co. KG).

### 2.6 Statistical analyses

Data were analyzed using PASW Statistics 19 and GraphPad. Prism 9. If the two groups of data conformed to a normal distribution, independent samples *t*-tests were used to investigate comparisons between two sets of data, and comparisons among three or more groups were made using one-way ANOVAs with Tukey’s tests. Differences were considered to be statistically significant if *p* < 0.05. The values given represent the mean ± standard deviation (SD).

## 3 Results

### 3.1 Structural identification of phenolic compounds one to five from *Thesium chinense*

**FIGURE 1:**
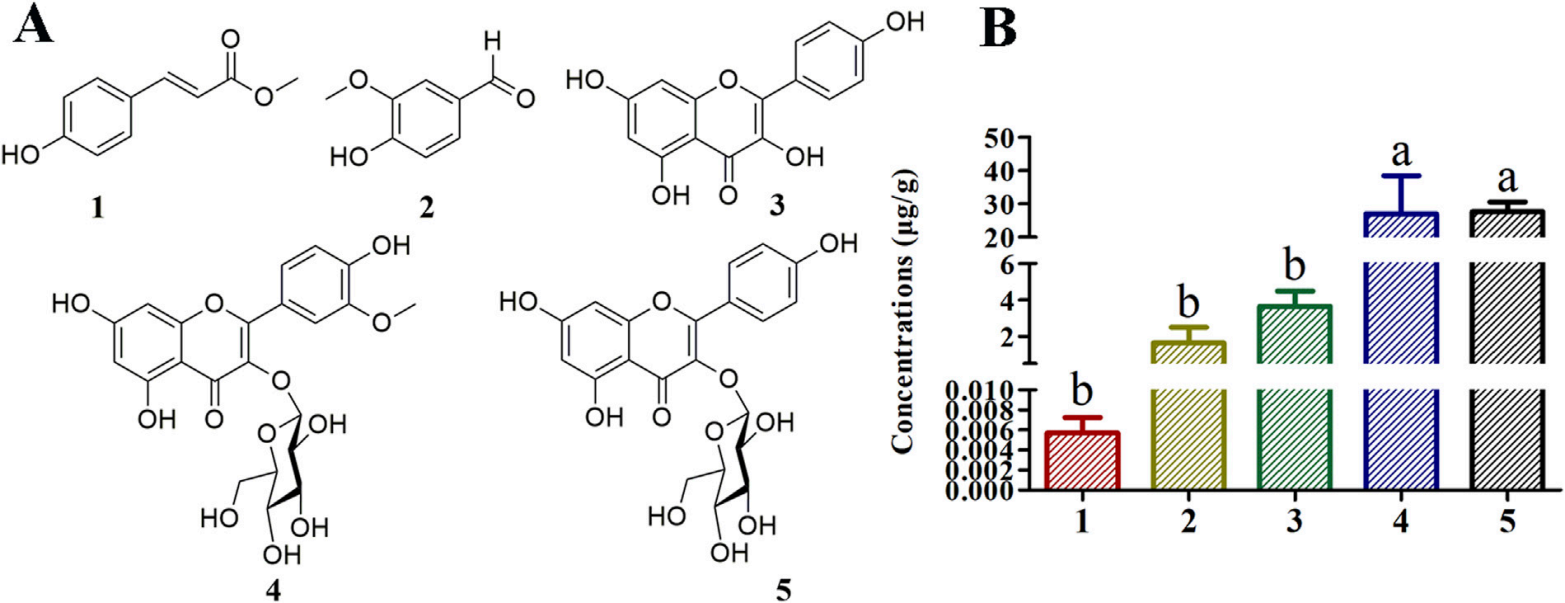
Structures of compounds isolated from *Thesium chinense* and quantitative analysis of their concentrations in Bairui Granules. **(A)** Chemical structures of flavonoid and phenolic compounds 1–5; **(B)** Concentrations of compounds one to five in Bairui Granules. Mean differences were compared using one-way ANOVA with Tukey’s test. The different small letters **(A, B)** represent significant differences at the *p* < 0.05 level. The results shown represent the means ± standard deviation.

Chemical separation and spectroscopic methods allowed the identification of compounds 1–5 as methyl-*p*-hydroxycinnamate (Kwon and Kim, 2003), vanillin (Pironti et al., 2021), kaempferol (Singh et al., 2008), isorhamnetin-3-*O*-glucoside (Fico et al., 2007), and astragalin (Wei et al., 2011), respectively (Supplementary Tables S3–S4; Figure 1A). Compounds 1 and 2 are phenolics, and compounds three to five are flavonoids. In this study compounds 2 and 4 have been isolated for the first time from *T. chinense*.

### 3.2 Qualitative and quantitative analyses of compounds one to five in Bairui Granules

**FIGURE 2:**
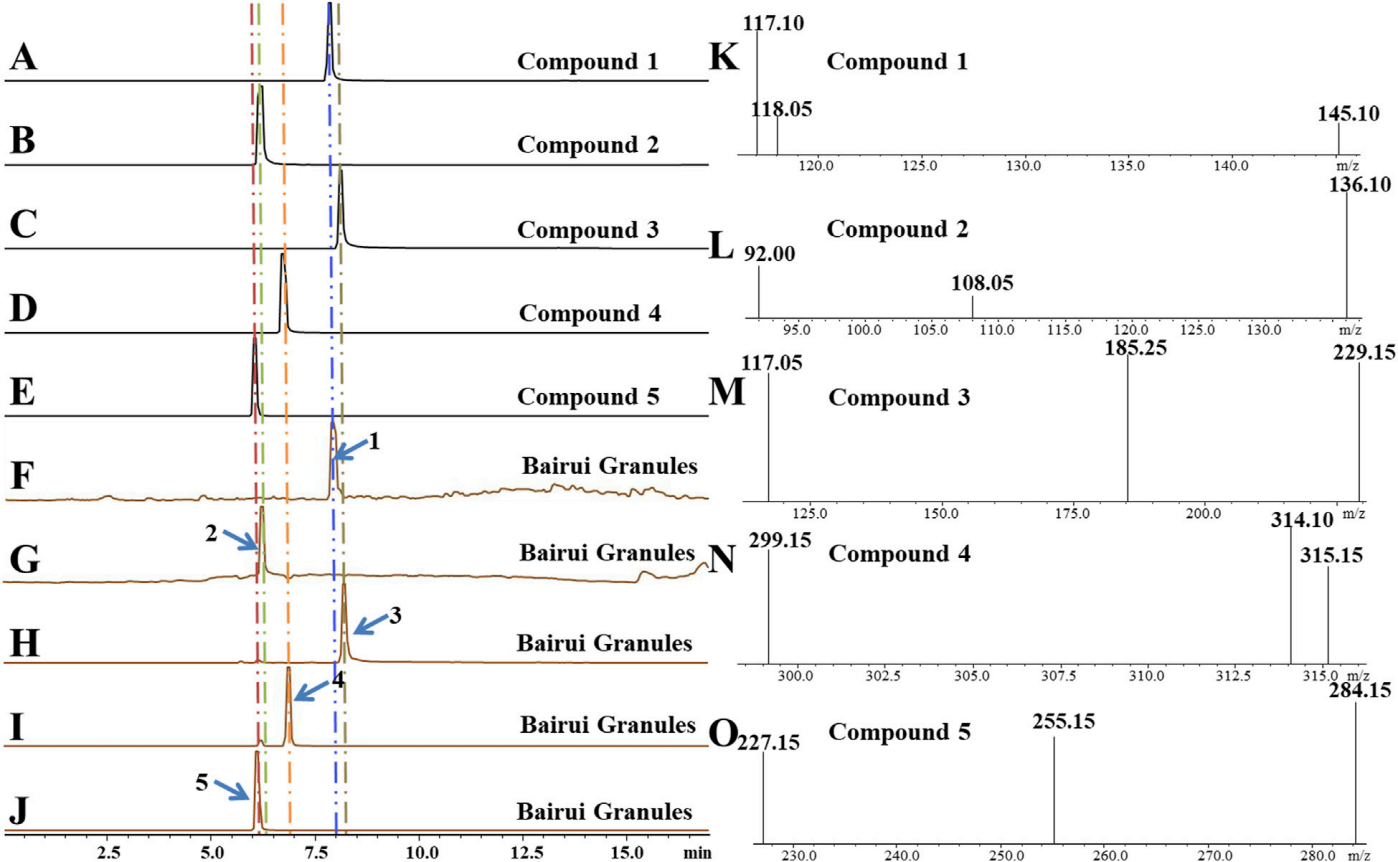
Qualitative analysis of in Bairui Granules using UPLC-MS/MS with an MRM-model. **(A-E)** Chromatogram of compounds 1–5; **(F-J)** Chromatograms of the Bairui Granules extracts; **(K-O)** Mass spectra of compounds one to five.

To determine concentrations of compounds one to five present in Bairui Granules, an extract of Bairui Granules was assessed using an UPLC-MS/MS. Prepared samples were all analyzed using an external standard. The retention times of compounds one to five were found to be 7.864, 6.215, 8.112, 6.772, and 6.068 min (Figure 2), respectively, and compounds one to five were present in Bairui Granules at concentrations of 0.006 ± 0.002, 1.63 ± 0.87, 3.65 ± 0.83, 26.97 ± 11.41, and 27.67 ± 2.91 *μ*g/g, respectively (Figure 1B, 2A–E). These results suggested that the concentrations of the flavonoid compounds three to five in Bairui Granules were higher than those of the phenolic compounds 1 and 2. This study represents that compounds 1, 2, and 4 have been detected for the first time in Bairui Granules.

### 3.3 Effect of compound 4 on the proliferation of CHO cells

**FIGURE 3:**
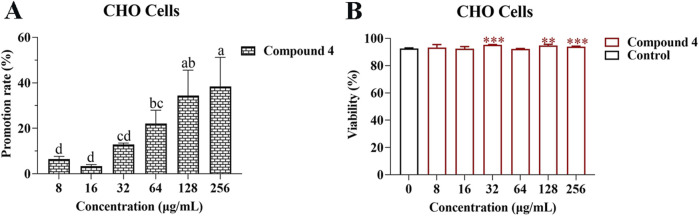
Effects of compound 4 on the proliferation and viability of CHO cells. **(A)** Promotion rate of compound 4 at different concentrations on the proliferation of CHO cells; **(B)** Effect of compound 4 at different concentrations on the viability of CHO cells. Mean differences were compared using one-way ANOVA with Tukey’s test. The different small letters **(A, B)** represent significant differences at the *p* < 0.05 level. Double or triple asterisks (** or ***) indicate significant differences between the control group and other treatments. ** Indicates *p* < 0.01. ***p < 0.001.

In order to determine the effect of compound 4 on the proliferation of CHO cells, different concentrations of compound 4 were co-cultured with CHO cells for 48 h. Examination with an inverted microscope revealed that after the CHO cells were treated with compound 4 at different concentrations, the cells remained round and bright, which was consistent with the morphology of the control group (Supplementary Figure S1). Treatment with compound 4 at concentrations of 8, 16, 32, 64, 128, and 256 *μ*g/mL led to increases in CHO cell proliferation, with the promotion rates being 6.46 ± 0.86, 3.44 ± 0.43, 12.91 ± 0.43, 22.09 ± 4.16, 34.43 ± 7.89, and 38.45% ± 9.04%, respectively (Figure 3A). Treatment with compound 4 at concentrations of 8, 16, and 64 *μ*g/mL had no obvious effect on CHO cells viability compared with the control. However, treatment with compound 4 at concentrations of 128 and 256 *μ*g/mL significantly promoted the viability of CHO cells (*p* < 0.01) (Figure 3B). The above results indicated that at the concentrations present in Bairui Granules, compound 4 was beneficial to the proliferation of CHO cells.

### 3.4 Effects of compounds 2, 4, and 5 on the proliferation of UCB NK cells

In order to investigate the effects of compounds 2, 4, and 5 on the proliferation of UCB NK cells, compounds 2, 4, and 5 at different concentrations were separately co-cultured with UCB NK cells for 48 h. UCB NK cell morphology was not affected under any concentrations of compounds 2, 4, or 5 (Supplementary Figures S2–S4).

When the concentrations of compound 2 were 16, 64, and 128 *μ*g/mL, the rate of promotion of UCB NK cell proliferation remained within a certain range (25.09% ± 0.56% to 28.21% ± 0.44%). However, at a concentration of 64 *μ*g/mL, compound 2 could significantly promote the viability of UCB NK cells compared with the control, while at a concentration of 128 *μ*g/mL, compound 2 exhibited a significant inhibitory effect.

At concentrations of 32, 64, 128, and 256 *μ*g/mL, compound 4 was able to promote the proliferation of UCB NK cells, with promotion rates ranging from 25.68% ± 0.02% to 70.81% ± 0.26%. However, at the same concentrations, compound 4 exhibited an inhibitory effect on the viability of UCB NK cells.

**FIGURE 4:**
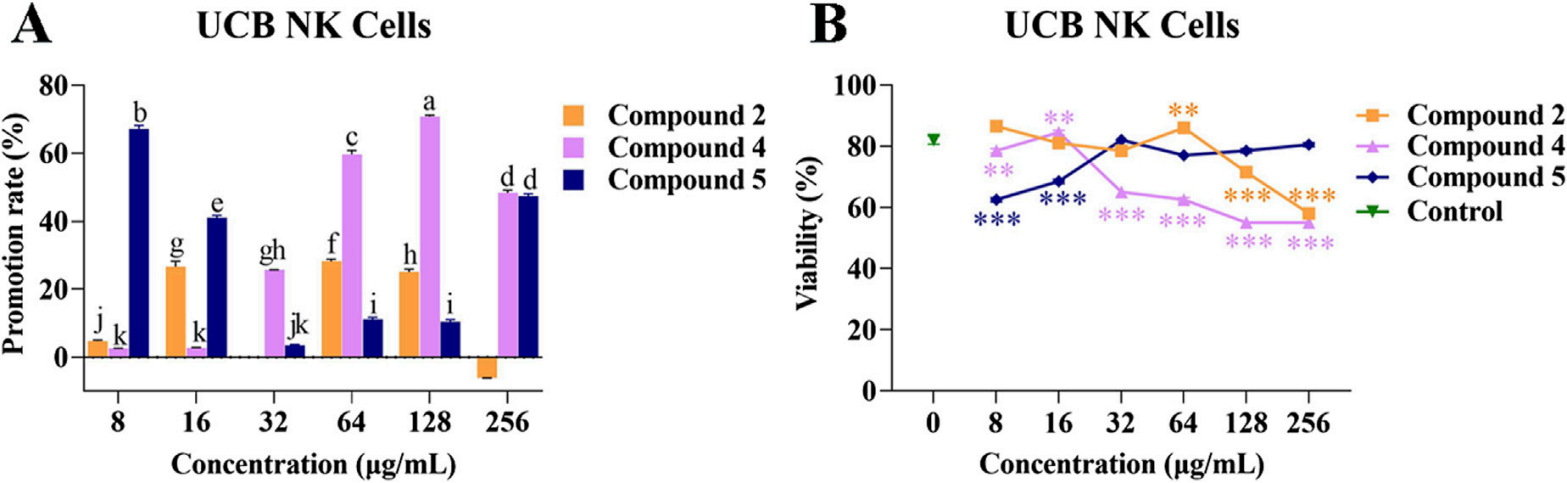
Effects of compounds 2, 4, and 5 on the proliferation and viability of UCB NK cells. **(A)** Promotion rate of compounds 2, 4, and 5 at different concentrations on the proliferation of UCB NK cells; **(B)** The effects of compounds 2, 4, and 5 at different concentrations on the viability of UCB NK cells. Mean differences were compared using one-way ANOVA with Tukey’s test. The different small letters (a, b, c, d, e, f, g, h, i, j, and k) represent significant differences at the *p* < 0.05 level. Double or triple asterisks (** or ***) indicate significant differences between the control group and other treatments. ** Indicates *p* < 0.01. ***p < 0.001.

When compound 5 was present at low concentrations (8 and 16 *μ*g/mL), the promotion rate of UCB NK cell proliferation ranged from 41.03% ± 0.48% to 67.22% ± 0.68%, but the compound exhibited an inhibitory effect on viability of the cells at these concentrations. At a concentration of 256 *μ*g/mL, compound 5 promoted cell proliferation, but had no effect on cell viability (Figures 4A, B). These results showed that the phenolic compound 2 was conducive to the proliferation of UCB NK cells while maintaining normal cell morphology and viability at a concentration of 64 *μ*g/mL.

### 3.5 The effects of compounds 1, 2, and 4 on the proliferation of PB NK cells

Compounds 1, 2, and 4 at different concentrations were co-cultured with PB NK cells to determine their effects on cell proliferation. Following co-culture for 48 h with compounds 1, 2, and 4 at different concentrations, no effect on PB NK cell morphology was observed (Supplementary Figures S5–S7). However, when the concentration of compound 1 was increased, the inhibition rate of PB NK cells proliferation also gradually increased, and the cell viability gradually decreased.

**FIGURE 5:**
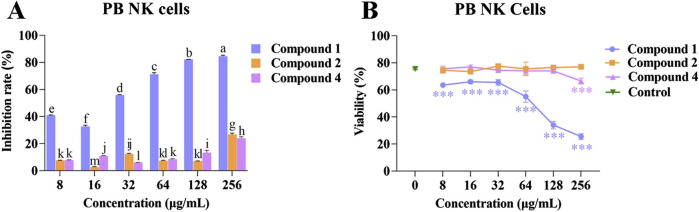
Effects of compounds 1, 2, and 4 on the proliferation and viability of PB NK cells. **(A)** Inhibition rate of compounds 1, 2, and 4 at different concentrations on the proliferation of PB NK cells; **(B)** The effects of compounds 1, 2, and 4 at different concentrations on the viability of PB NK cells. Mean differences were compared using one-way ANOVA with Tukey’s test. The different small letters (a, b, c, d, e, f, g, h, i, j, k, l, and m) represent significant differences at the *p* < 0.05 level. Triple asterisks (***) indicate significant differences between the control group and other treatments. ***p < 0.001.

The inhibitory rate of compound 2 on the proliferation of PB NK cells (2.70% ± 0.22% to 26.80% ± 0.68%) was comparable to that of compound 4 (6.08% ± 0.04% to 23.96% ± 0.80%). Compounds 2 and 4 at other concentrations had no effect on viability of PB NK cells compared with the control, although at a concentration of 256 *μ*g/mL, compound 4 had an inhibitory effect on cell viability (Figures 5A, B). The above results suggested that compound 1 has a higher inhibitory effect on the proliferation of PB NK cells than compounds 2 and 4.

### 3.6 The effects of compounds 1, 2, and 4 on the proliferation of UCB MSC cells

Compounds 1, 2, and 4 at different concentrations were co-cultured with UCB MSC cells to investigate their effects on cell proliferation. Following co-culture for 48 h with compounds 1, 2, and 4 at different concentrations, the UCB MSC cells were spindle shaped, which is consistent with normal MSC cell morphology (Supplementary Figures S8–S10). The promotion rates of compounds 1 and 4 at different concentrations on UCB MSC cell proliferation range from 1.64% ± 0.19% to 11.28% ± 0.16% and from 1.66% ± 0.05% to 27.64% ± 0.51%, respectively.

**FIGURE 6:**
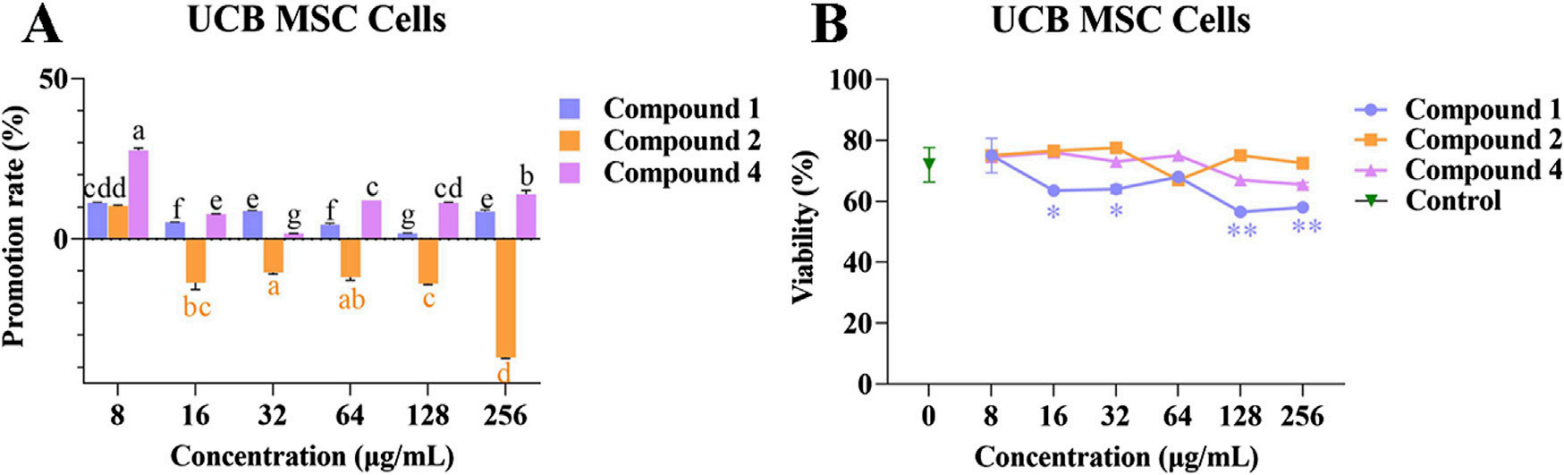
The effects of compounds 1, 2, and 4 on the proliferation and viability of UCB MSC cells. **(A)** Effect of compounds 1, 2, and 4 at different concentrations on the proliferation of UCB MSC cells; **(B)** The effects of compounds 1, 2, and 4 at different concentrations on the viability of UCB MSC cells. Mean differences were compared using one-way ANOVA with Tukey’s test. The different small letters (a, b, c, d, e, f, and g) represent significant differences at the *p* < 0.05 level. Single or double asterisks (* or **) indicate significant differences between the control group and other treatments. *p < 0.05. ** Indicates *p* < 0.01.

Compound 2 inhibited UCB MSC cell proliferation at concentrations of 16, 32, 128, and 256 *μ*g/mL, but had a promoting effect at a concentration of 8 *μ*g/mL (Figure 6A). Compared with the control, compound 1 had a significant inhibitory effect on UCB MSC cell viability at concentrations of 16, 32, 128, and 256 *μ*g/mL (*p* < 0.05), while compounds 2 and 4 had no significant effect on cell viability (Figure 6B). The above results indicate that compound 4 is able to promote UCB MSC cells proliferation without affecting cell viability.

### 3.7 The effects of compounds 1, 2, and 4 on the expression of PB NK cell markers

**FIGURE 7:**
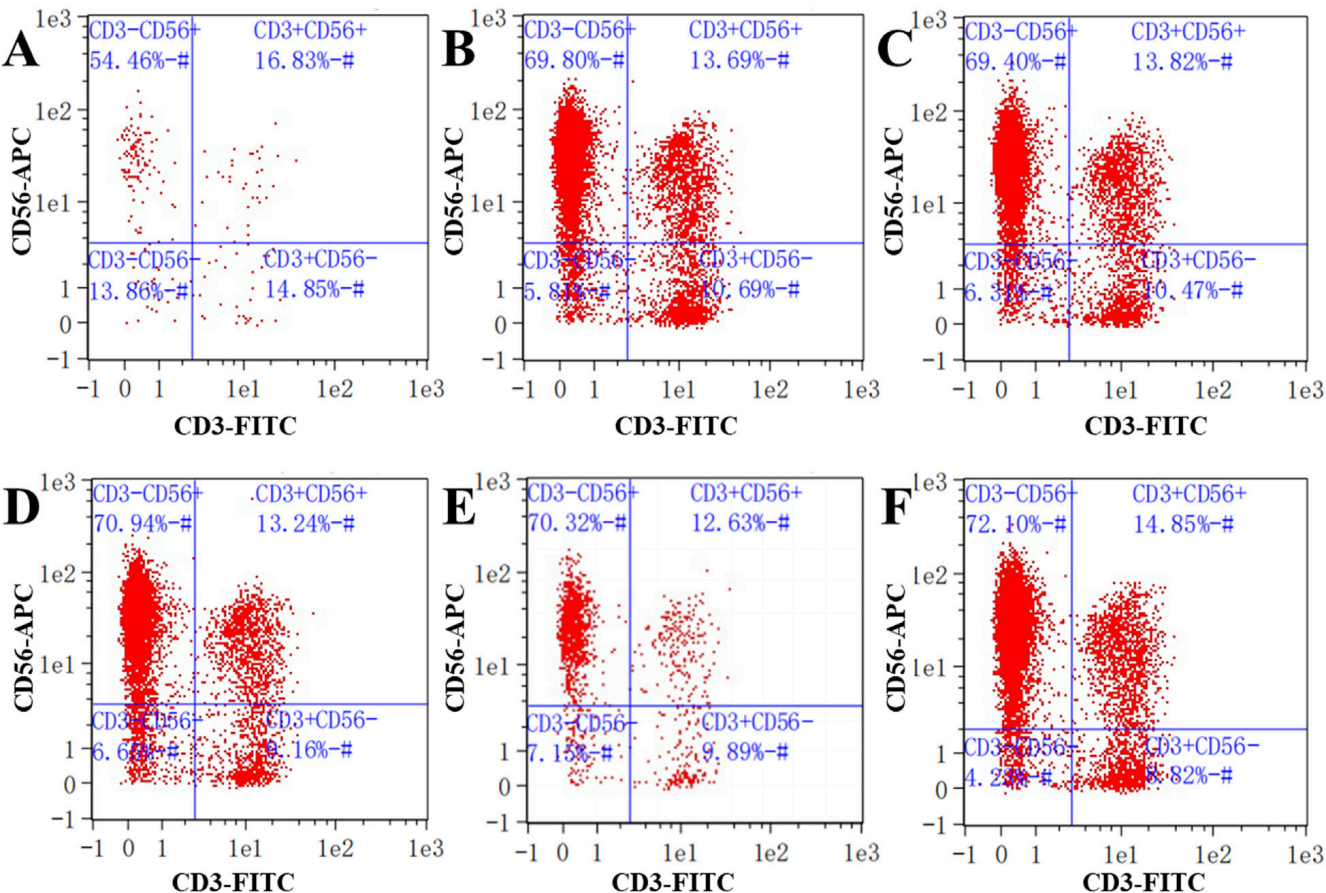
Detection of NK cell surface markers. **(A)** Expression of NK cell biomarkers in cells co-cultured with compound 1 at a concentration of 32 *μ*g/mL; **(B, C)** Expression of NK cell biomarkers in cells co-cultured with compound 2 at concentrations of 8 and 16 *μ*g/mL; **(D, E)** Expression of NK cell biomarkers in cells co-cultured with compound 4 at concentrations of 8 and 16 *μ*g/mL; **(F)** Expression of NK cell biomarkers in the control. CD3^−^represents CD3 negative; CD56^+^ represents CD56 positive.

We next wanted to investigate the effects of low concentrations of compounds 1, 2, and 4 on the expression of NK cell markers. Low concentrations of compounds 1, 2, and 4 were co-cultured separately with PB NK cells. In human pathology, the proportion of CD3^−^CD56^+^ is commonly used as a proxy for NK cells in the diagnosis of certain diseases. The proportion of NK cells with a CD3^−^CD56^+^ cell phenotype when cultured in the presence of compound 1 at a concentration of 32 *μ*g/mL was 54.46% (Figure 7A). Similarly, after 48 h of co-culture of PB NK cells with compound 2 at concentrations of 8 and 16 *μ*g/mL, the proportion of cells with the CD3^−^CD56^+^ phenotype were 69.80% and 69.40%, respectively, while the proportions of cells co-cultured with this phenotype following co-culture with compound 4 under the same conditions were 70.94% and 70.32%, respectively (Figures 7B–E). These results suggest that compound 1 had an inhibitory effect on PB NK cell differentiation compared to the control (72.10%) at a concentration of 32 *μ*g/mL.

## 4 Discussion

### 4.1 Bairui granules contain a high diversity of phenolic compounds

*T. chinense* is an important traditional Chinese medicine. When applied as a crude drug, *T. chinense* is able to significantly reduce ear swelling caused by xylene in mice (Yuan et al., 2006). Moreover, a concentration of the methanol extract of *T. chinense* is able to significantly reduce the edema in mouse paws induced by carrageenan, and reduces xylene-induced ear edema in mice (Parveen et al., 2007). The chemical substances present in this plant have been widely studied and identified. At present, most of the compounds isolated from *T. chinense* have been flavonoids and phenylpropanoids (Li G. H. et al., 2021; Liu et al., 2023). In this study, we isolated and identified two phenolic compounds and three flavonoids from *T. chinense*, with vanillin (2) and isorhamnetin-3-*O*-glucoside (4) isolated from this plant for the first time here. The concentrations of these compounds in Bairui Granules were further determined using UPLC-MS/MS.

### 4.2 Phenolic compounds exhibit diverse biological activities

Flavonoids in *T. chinense* have abundant biological activity. Previous research on the activities of kaempferol (3) and astragalin (5) mainly focused on their anti-inflammatory and analgesic properties (Ramanan et al., 2016; Sudarshan and Aidhen, 2016). Kaempferol (3) inhibited the writhing reaction following abdominal contraction tests induced by a certain concentration of acetic acid in mice (Parveen et al., 2007). Certain concentrations of astragalin (5) were able to inhibit the infiltration of inflammatory cells, reduce breast congestion and edema and inhibit the release of inflammatory cytokines in a mastitis mouse model (Li et al., 2013). Moreover, astragalin (5) was also able to block the NF-κB signaling pathway to combat colitis (Han et al., 2020). Diverse biological activities have also been observed in methyl-*p*-hydroxycinnamate (1), vanillin (2), and isorhamnetin-3-*O*-glucoside (4). Methyl-*p*-hydroxycinnamate (1) was found to inhibit inflammation of RAW 264.7 macrophages under LSP induced stimulation (Vo et al., 2014). Vanillin (2) and isorhamnetin-3-*O*-glucoside (4) both had anticancer, anti-inflammatory, and antibacterial effects. Furthermore, vanillin (2) also has a protective effect on nerves and blood (Arya et al., 2021; Du et al., 2014). From this, it can be seen that research into *T. chinense* mainly focuses on how the component chemical substances exert their therapeutic effects. However, Bairui Granules are a medical preparation, whose only herbal ingredient is *T. chinense*, and to date there has been little research into the effects of the active substances on mammalian expression vectors, human immune cells, and stem cells.

### 4.3 Phenolics are the most biologically active substances in Bairui Granules

Flavonoids and phenolic compounds have certain effects on mammalian expression vectors, human immune cells, and stem cells (Burkard et al., 2017; Ozdal et al., 2018). Flavonoids from *Astragalus complanatus* R.Br. ex Bunge. seeds significantly promoted the proliferation of NK cells (Han et al., 2015), and the flavonoid quercetin has a close relationship with the differentiation of MSC cells (Yu et al., 2010). In addition, phenolic compounds are able to increase NK cell activity significantly and can also serve as antioxidants to protect MSC cells (Kilani Jaziri et al., 2016; Li H. S. et al., 2021). In this study, the methyl-*p*-hydroxycinnamate (1), vanillin (2), and isorhamnetin-3-*O*-glucoside (4) present in Bairui Granules were qualitatively and quantitatively studied for the first time. Importantly, isorhamnetin-3-*O*-glucoside (4) was able to promote the proliferation of CHO cells, UCB NK cells, and UCB MSC cells when its concentration was close to that found in the Bairui Granules. Meanwhile, methyl-*p*-hydroxycinnamate (1) was conducive to the proliferation of UCB MSC cells, and vanillin (2) and astragalin (5) at different concentrations all promoted the proliferation of UCB NK cells. These results indicated that methyl-*p*-hydroxycinnamate (1), vanillin (2), isorhamnetin-3-*O*-glucoside (4), and astragalin (5) were potentially the active substances in Bairui Granules. However, not all flavonoids and phenolic compounds promote the activity of immune cells (Perche et al., 2014). Our results also demonstrated that methyl-*p*-hydroxycinnamate (1), vanillin (2), and isorhamnetin-3-*O*-glucoside (4) exhibited inhibitory effects on PB NK cells. The cytotoxicity tests of the phenolic compounds present in Bairui Granules could thus provide a scientific basis for the medicinal value of *T. chinense*.

## 5 Conclusion

Five phenolics were identified in Bairui Granules that obtained from the medicinal plant *T. chinense.* Moreover, methyl-*p*-hydroxycinnamate (1), vanillin (2), and isorhamnetin-3-*O*-glucoside (4) were found for the first time in Bairui Granules. These metabolites had a certain effect on mammalian expression vectors, human immune cells, and stem cells. The biological activity of those secondary metabolites from *T. chinense* justify the medicinal use of this plant.

## Data Availability

The data presented in the study are deposited in the figshare repository, accession number 10.6084/m9.figshare.27894771.
