# Supplementary material for: Cytotoxic activity of phenolic compounds in Bairui Granules obtained from the Chinese medicinal plant Thesium chinense
Source: Front Chem. 2024 Dec 2;12:1506792. doi: 10.3389/fchem.2024.1506792 (PMC11646769; doi:10.3389/fchem.2024.1506792)
Supplement: Supplementary file 1 [file DataSheet1.docx]

**Cytotoxic activity of phenolic compounds in Bairui Granules obtained from the Chinese medicinal plant *Thesium chinense***

**Supporting Information**

Shaobin Zhang, Hong Chen, Juan Hua*, and Shihong Luo*

Engineering Research Center of Protection and Utilization of Plant Resources, College of Bioscience and Biotechnology, Shenyang Agricultural University, Shenyang, 110866, Liaoning Province, China

*Corresponding Authors

Juan Hua, huajuan@syau.edu.cn; Prof. Shihong Luo, E-mail address: luoshihong@syau.edu.cn (http://orcid.org/0000-0003-3500-3466)

**Table of Contents**

**[Table S1](#_Toc493862345)**[. Specific MRM parameters for compounds](#_Toc493862345) **[1−5](#_Toc493862345)** [S3](#_Toc493862345)

**[Table S2](#_Toc493862347)**[. UPLC-MS/MS calibration curves for compounds](#_Toc493862347) **[1−5](#_Toc493862347)** [S4](#_Toc493862347)

**Table S3**. ^1^H-NMR and ^13^C-NMR data of compounds **1**, **2**, and **3** S5

**[Table S4.](#_Toc493862348)**^[1](#_Toc493862348)^[H-NMR and](#_Toc493862348) ^[13](#_Toc493862348)^[C-NMR data of compounds](#_Toc493862348) **[4](#_Toc493862348)** [and](#_Toc493862348) **[5](#_Toc493862348)** [S6](#_Toc493862348)

**[Figure S1](#_Toc493862348)**[. Effect of compound](#_Toc493862348) **[4](#_Toc493862348)** [on the morphology of CHO cells at concentrations 256, 128, 64, 32, 16, and 8](#_Toc493862348) *[μ](#_Toc493862348)*[g/mL S7](#_Toc493862348)

**[Figure S2](#_Toc493862348)**[. Effect of compound](#_Toc493862348) **[2](#_Toc493862348)** [on the morphology of UCB NK cells at concentrations of 256, 128, 64, 32, 16, and 8](#_Toc493862348) *[μ](#_Toc493862348)*[g/mL . S8](#_Toc493862348)

**[Figure S3](#_Toc493862349)**[. Effect of compound](#_Toc493862349) **[4](#_Toc493862349)** [on the morphology of UCB NK cells at concentrations of 256, 128, 64, 32, 16, and 8](#_Toc493862349) *[μ](#_Toc493862349)*[g/mL . S9](#_Toc493862349)

**[Figure S4](#_Toc493862349)**[. Effect of compound](#_Toc493862349) **[5](#_Toc493862349)** [on the morphology of UCB NK cells at concentrations of 256, 128, 64, 32, 16, and 8](#_Toc493862349) *[μ](#_Toc493862349)*[g/mL . S10](#_Toc493862349)

**Figure S5.** Effect of compound **1** on the morphology of PB NK cells at concentrations of 256, 128, 64, 32, 16, and 8 *μ*g/mL [S11](#_Toc493862351)

**Figure S6.** Effect of compound **2** on the morphology of PB NK cells at concentrations of 256, 128, 64, 32, 16, and 8 *μ*g/mL  [S12](#_Toc493862351)

**Figure S7.** Effect of compound **4** on the morphology of PB NK cells at concentrations of 256, 128, 64, 32, 16, and 8 *μ*g/mL[. S13](#_Toc493862351)

**Figure S8.** [Effect of compound](#_Toc493862351) **[1](#_Toc493862351)** [on the morphology of UCB MSC cells at concentrations of 256, 128, 64, 32, 16, and 8](#_Toc493862351) *[μ](#_Toc493862351)*[g/mL S14](#_Toc493862351)

**Figure S9.** [Effect of compound](#_Toc493862351) **[2](#_Toc493862351)** [on the morphology of UCB MSC cells at concentrations of 256, 128, 64, 32, 16, and 8](#_Toc493862351) *[μ](#_Toc493862351)*[g/mL S15](#_Toc493862351)

**Figure S10.** Effect of compound **4** on the morphology of UCB MSC cells at concentrations of 256, 128, 64, 32, 16, and 8 μg/mL[. S16](#_Toc493862351)

**Table S1.** Specific MRM parameters for compounds **1−5**.

| **No.** | **Compound** | **Polarity** | **Precursor ion** | **Product ion** | **Q1 Pre Bias (V)** | **CE (V)** | **Q3 Pre Bias (V)** |
| --- | --- | --- | --- | --- | --- | --- | --- |
| **1** | **Methyl-*p*-hydroxycinnamate** | Negative | 177.00 | 117.10 | 12.0 | 29.0 | 10.0 |
|  |  |  |  | 118.05 | 12.0 | 19.0 | 10.0 |
|  |  |  |  | 145.10 | 13.0 | 20.0 | 13.0 |
| **2** | **Vanillin** | Negative | 151.00 | 136.10 | 10.0 | 18.0 | 24.0 |
|  |  |  |  | 92.00 | 10.0 | 22.0 | 27.0 |
|  |  |  |  | 108.05 | 10.0 | 23.0 | 16.0 |
| **3** | **Kaempferol** | Negative | 285.15 | 229.15  117.05  182.25 | 20.0  18.0  19.0 | 25.0  43.0  28.0 | 23.0  10.0  17.0 |
| **4** | **Isorhamnetin-3-glucoside** | Negative | 477.05 | 314.10 | 11.0 | 27.0 | 13.0 |
|  |  |  |  | 299.15 | 13.0 | 40.0 | 19.0 |
|  |  |  |  | 315.15 | 11.0 | 24.0 | 14.0 |
| **5** | **Astragalin** | Negative | 447.00 | 284.15 | 16.0 | 28.0 | 18.0 |
|  |  |  |  | 255.15 | 16.0 | 41.0 | 15.0 |
|  |  |  |  | 227.15 | 16.0 | 48.0 | 22.0 |

**Table S2.** UPLC-MS/MS calibration curves for compounds **1−5**.

| **No.** | **Compound** | **Calibration curve** | **R^2^** |
| --- | --- | --- | --- |
| **1** | **Methyl-p-hydroxycinnamate** | Y=(1.4909×10^-7^)X+0.0636 | 0.9993 |
| **2** | **Vanillin** | Y=(2.1123×10^-6^)X+0.0248 | 0.9985 |
| **3** | **Kaempferol** | Y=(4.2873×10^-6^)X-0.0081 | 0.9954 |
| **4** | **Isorhamnetin-3-glucoside** | Y=(1.7689×10^-6^)X+0.1095 | 0.9957 |
| **5** | **Astragalin** | Y=(1.7909×10^-7^)X+0.0571 | 0.9974 |

**Table S3.** ^1^H-NMR and ^13^C-NMR data pertaining to compounds **1**–**3**.

| Position | **1** (acetone-*d*_6_) | | **2** (acetone-*d*_6_) | | **3** (methanol-*d*_4_) | |
| --- | --- | --- | --- | --- | --- | --- |
|  | *δ*_H_ (multi, *J* in Hz) | *δ*_C_ | *δ*_H_ (multi, *J* in Hz) | *δ*_C_ | *δ*_H_ (multi, *J* in Hz) | *δ*_C_ |
| 1 | - | 167.9, s | 9.81 (s) | 191.1, d | - | - |
| 2 | 6.33 (dd, 1.9, 15.9) | 115.2, d | - | 130.6, s | - | 148.0, s |
| 3 | 7.59 (d, 15.9) | 145.4, d | - | 110.9, d | - | 137.2, s |
| 4 | - | 126.8, s | - | 149.0, s | - | 177.4, s |
| 5 | 7.53 (m) | 130.9, d | - | 153.6, s | - | 162.5, s |
| 6 | 6.87 (dd, 6.9, 13.6) | 116.7, d | 7.00 (d, 7.9) | 115.9, d | 6.17 (s) | 99.3, d |
| 7 | - | 160.6, s | 7.44 (d, 4.9) | 127.0, d | - | 165.6, s |
| 8 | 6.87 (dd, 6.9, 13.6) | 116.7, d | - | - | 6.38 (s) | 94.4, d |
| 9 | 7.53 (m) | 130.9, t | - | - | - | 158.2, s |
| 10 | - | - | - | - | - | 104.5, s |
| OMe | 3.71 (s) | 51.5, t | 3.92 (s) | 56.2, t | - | - |
| 1' | - | - | - | - | - | 123.7, s |
| 2' | - | - | - | - | 8.07 (d, 8.2) | 130.7, d |
| 3' | - | - | - | - | 6.89(d, 8.4) | 116.3, d |
| 4' | - | - | - | - | - | 160.5, s |
| 5' | - | - | - | - | 6.89(d, 8.4) | 116.3, d |
| 6' | - |  | - | - | 8.07 (d, 8.2) | 130.7, d |

^1^H NMR spectrum data of compounds **1**–**3** were recorded at 600 MHz, and ^13^C NMR spectrum data of compounds **1**–**3** were recorded at 150 MHz.

**Table S4.** ^1^H and ^13^C NMR data pertaining to compounds **4** and **5**.

| Position | **4** (methanol-*d*_4_) | | **5** (dimethylsulfoxide-*d*_6_) | |
| --- | --- | --- | --- | --- |
|  | *δ*_H_ (multi, *J* in Hz) | *δ*_C_ | *δ*_H_ (multi, *J* in Hz) | *δ*_C_ |
| 2 | - | 150.0, s | - | 156.6, s |
| 3 | - | 130.6, s | - | 133.4, s |
| 4 | - | 179.9, s | - | 177.7, s |
| 5 | - | 159.4, s | - | 160.2, s |
| 6 | 6.34 (d, 1.9) | 99.1, d | 6.17 (d, 1.9) | 99.2, d |
| 7 | - | 167.4, s | - | 161.5, s |
| 8 | 6.60 (d, 1.8) | 93.1, d | 6.40 (d, 1.9) | 94.1, d |
| 9 | - | 158.4, s | - | 156.8, s |
| 10 | - | 116.6, s | - | 104.1, s |
| OMe | 3.88 (s) | 56.5, q | - | - |
| 1' | - | 123.0, s | - | 121.2, s |
| 2' | 7.72 (d, 1.9) | 123.2, d | 8.00 (d, 8.9) | 131.2, d |
| 3' | 7.61(dd, 2.0, 8.4) | 117.6, d | 6.86 (d, 8.9) | 115.5, d |
| 4' | - | 135.8, s | - | 165.0, s |
| 5' | - | 146.0, s | 6.86 (d, 8.9) | 115.5, d |
| 6' | 6.87 (d, 8.4) | 116.0, d | 8.00 (d, 8.9) | 131.2, d |
| 3-Glc | | | 3-Glc | |
| 1 | 5.30 (d, 7.7) | 104.0, d | 5.40 (d, 7.6) | 101.2, d |
| 2 | 3.71 (d, 11.9) | 78.5, d | 3.18 (m) | 74.5, d |
| 3 | 3.43 (d, 8.9) | 78.1,d | 3.18 (m) | 76.6, d |
| 4 | 3.22 (m) | 71.2, d | 3.18 (m) | 70.1, d |
| 5 | 3.48 (m) | 75.7, d | 3.18 (m) | 77.7, d |
| 6 | 3.57 (m) | 62.5, t | 3.18 (m) | 61.1, t |

^1^H NMR spectrum data of compounds **4** and **5** were recorded at 600 MHz, and ^13^C NMR spectrum data of compounds **4** and **5** were recorded at 150 MHz.


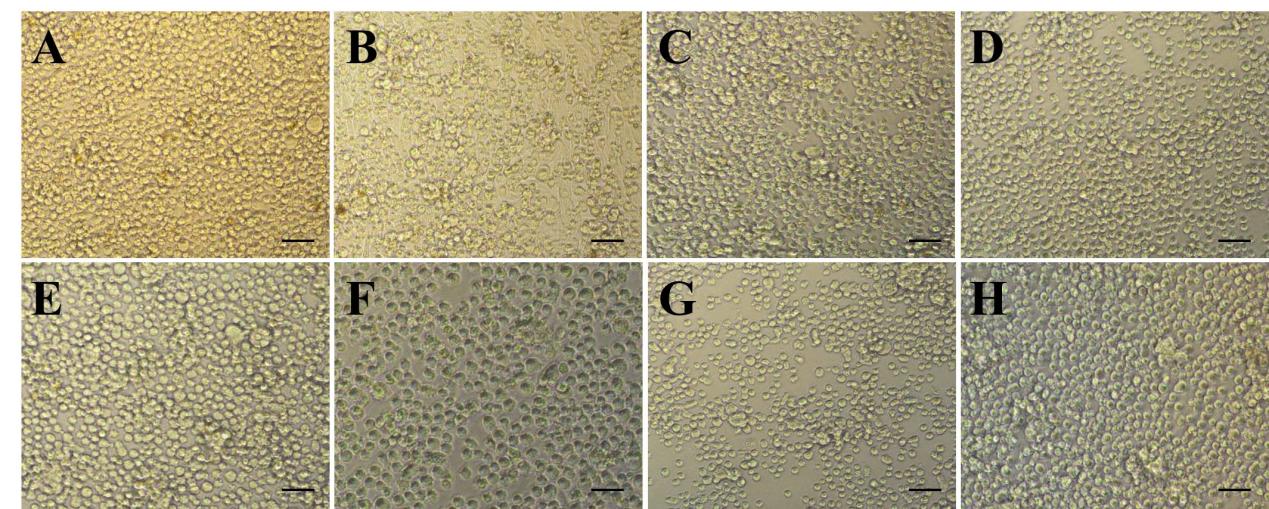


**Figure S1.** The effect of compound **4** on the morphology of CHO cells at concentrations of 256 (A), 128 (B), 64 (C), 32 (D), 16 (E), and 8 (F) *μ*g/mL, viewed under an eyepiece (10×) and an objective lens (20×), following co-culture for 48 h. Cell morphology of the negative control (G) and the blank control (H). Scale bar = 100 *μ*m.


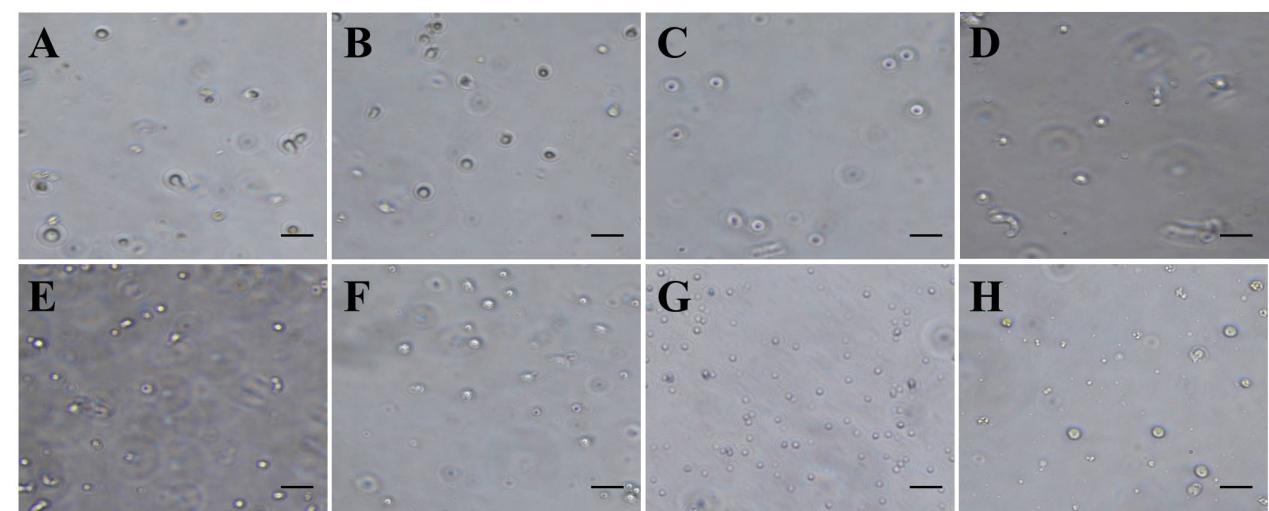


**Figure S2.** The effect of compound **2** on the morphology of UCB NK cells at concentrations of 256 (A), 128 (B), 64 (C), 32 (D), 16 (E), and 8 (F) *μ*g/mL, viewed under an eyepiece (10×) and an objective lens (20×), following co-culture for 48 h. Cell morphology of the negative control (G) and the blank control (H). Scale bar = 100 *μ*m.


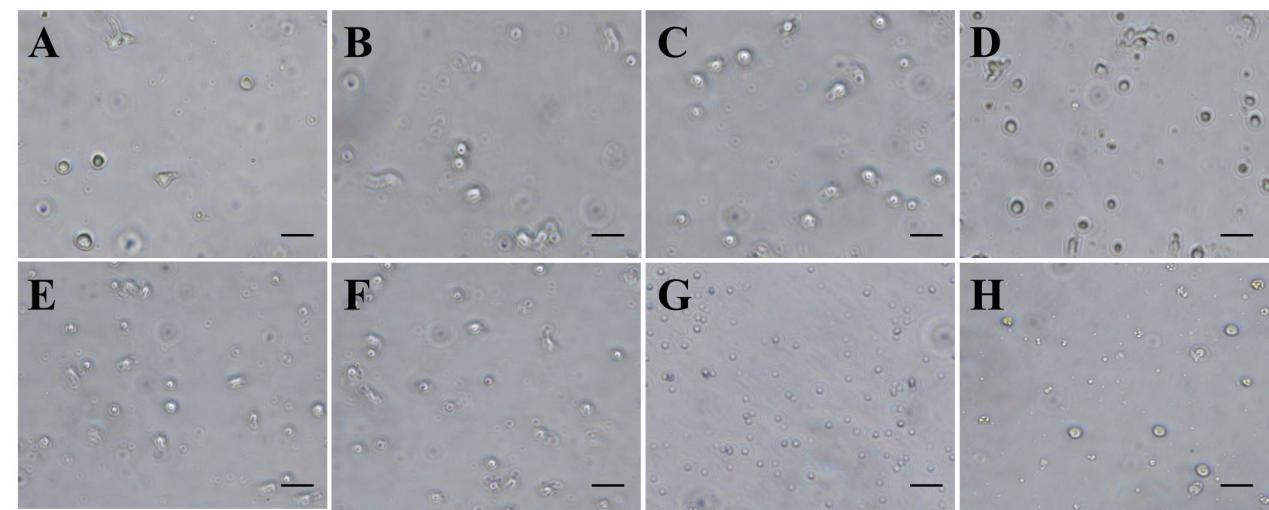


**Figure S3.** The effect of compound **4** on the morphology of UCB NK cells at concentrations of 256 (A), 128 (B), 64 (C), 32 (D), 16 (E), and 8 (F) *μ*g/mL, viewed under an eyepiece (10×) and an objective lens (20×), following co-culture for 48 h. Cell morphology of the negative control (G) and the blank control (H). Scale bar = 100 *μ*m.


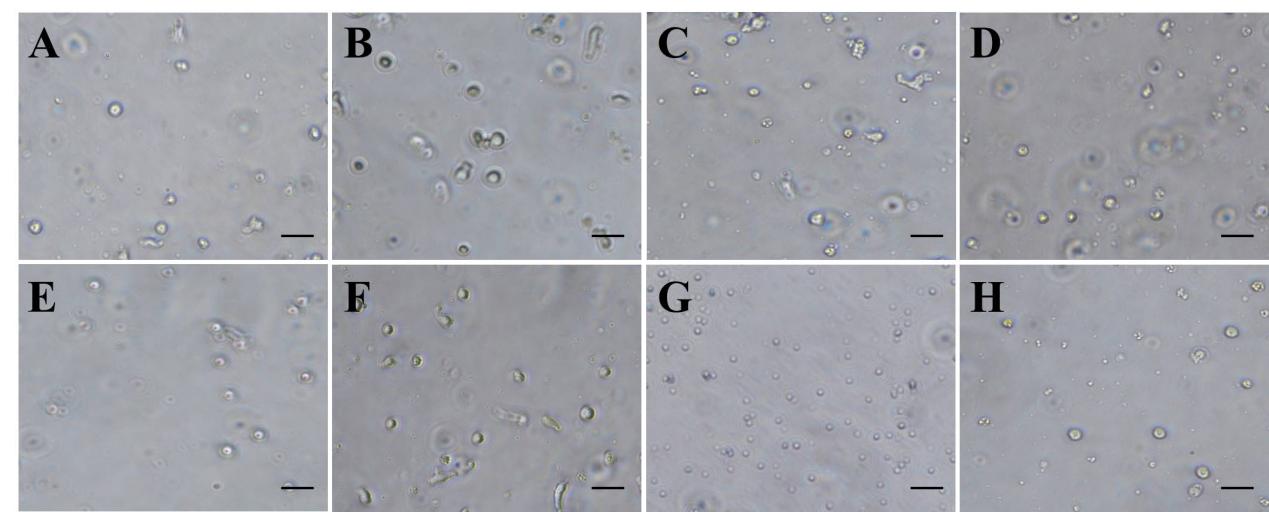


**Figure S4.** The effect of compound **5** on the morphology of UCB NK cells at concentrations of 256 (A), 128 (B), 64 (C), 32 (D), 16 (E), and 8 (F) *μ*g/mL, viewed under an eyepiece (10×) and an objective lens (20×), following co-culture for 48 h. Cell morphology of the negative control (G) and the blank control (H). Scale bar = 100 *μ*m.


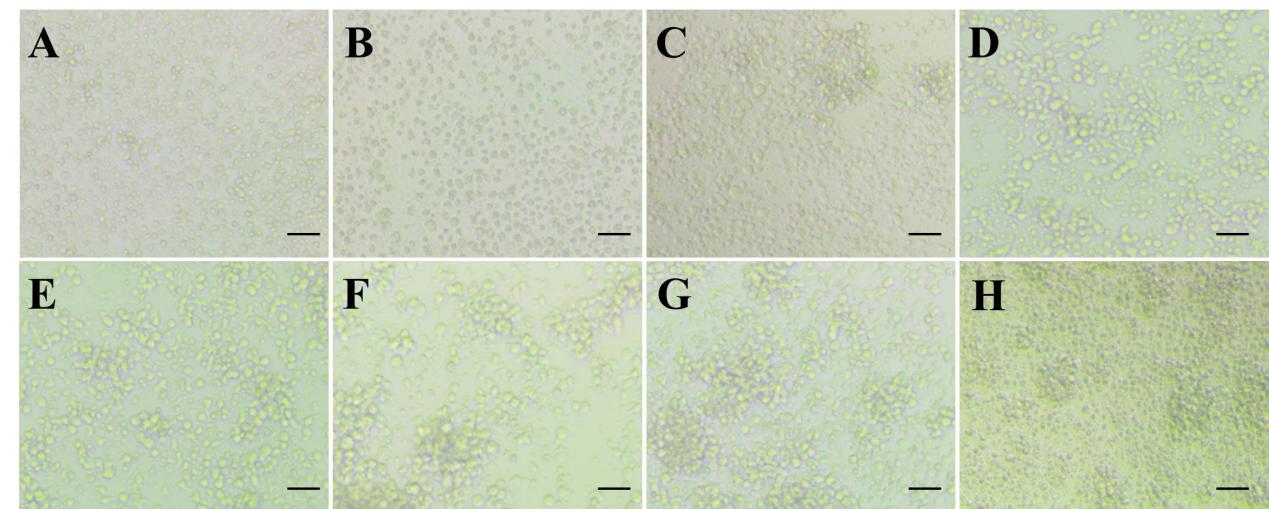


**Figure S5.** The effect of compound **1** on the morphology of PB NK cells at concentrations of 256 (A), 128 (B), 64 (C), 32 (D), 16 (E), and 8 (F) *μ*g/mL, viewed under an eyepiece (10×) and an objective lens (20×), following co-culture for 48 h. Cell morphology of the negative control (G) and the blank control (H). Scale bar = 100 *μ*m.


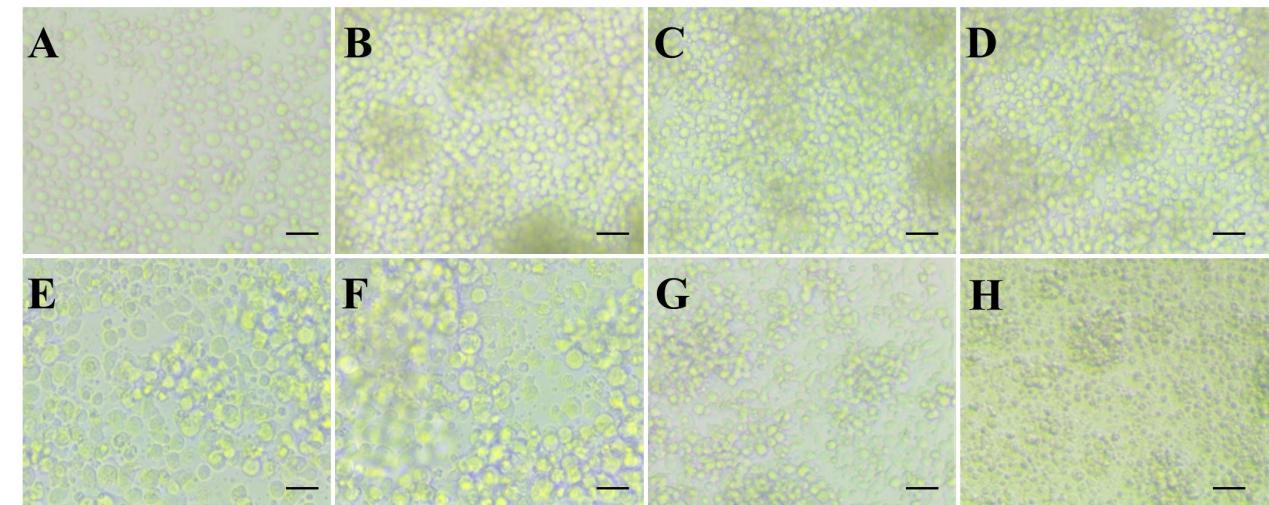


**Figure S6.** The effect of compound **2** on the morphology of PB NK cells at concentrations of 256 (A), 128 (B), 64 (C), 32 (D), 16 (E), and 8 (F) *μ*g/mL, viewed under an eyepiece (10×) and an objective lens (20×), following co-culture for 48 h. Cell morphology of the negative control (G) and the blank control (H). Scale bar = 100 *μ*m.


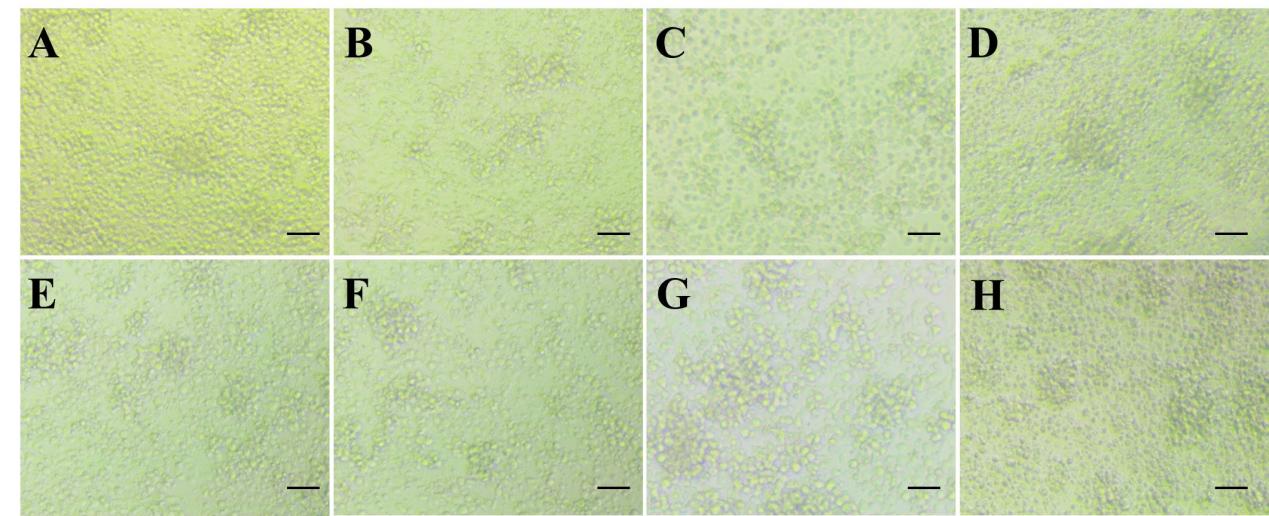


**Figure S7.** The effect of compound **4** on the morphology of PB NK cells at concentrations of 256 (A), 128 (B), 64 (C), 32 (D), 16 (E), and 8 (F) *μ*g/mL, viewed under an eyepiece (10×) and an objective lens (20×), following co-culture for 48 h. Cell morphology of the negative control (G) and the blank control (H). Scale bar = 100 *μ*m.


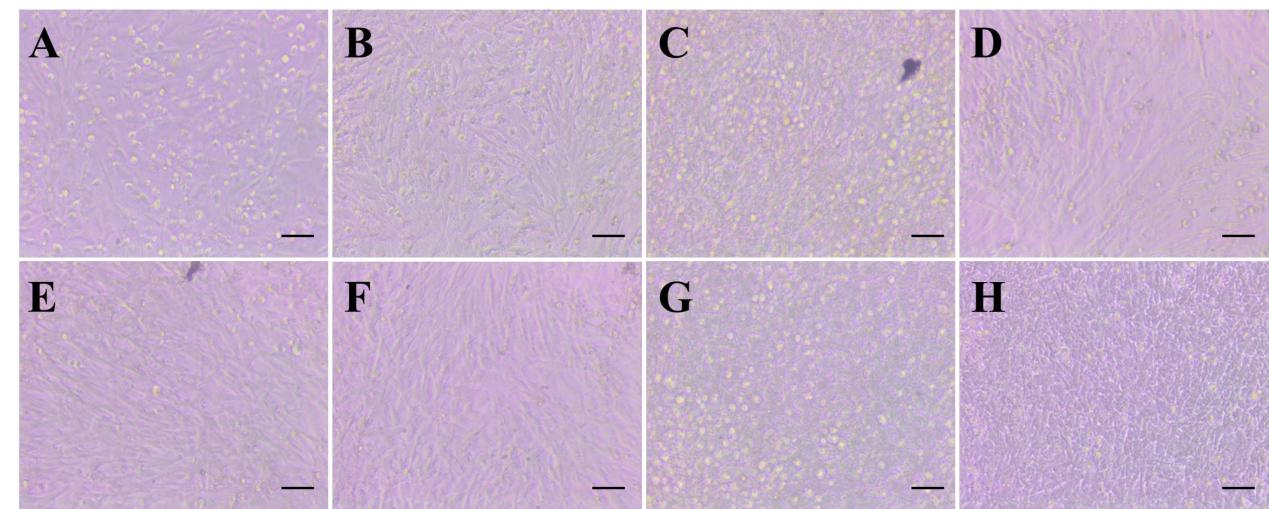


**Figure S8.** The effect of compound **1** on the morphology of UCB MSC cells at concentrations of 256 (A), 128 (B), 64 (C), 32 (D), 16 (E), and 8 (F) *μ*g/mL, viewed under an eyepiece (10×) and an objective lens (20×), following co-culture for 48 h. Cell morphology of the negative control (G) and the blank control (H). Scale bar = 100 *μ*m.


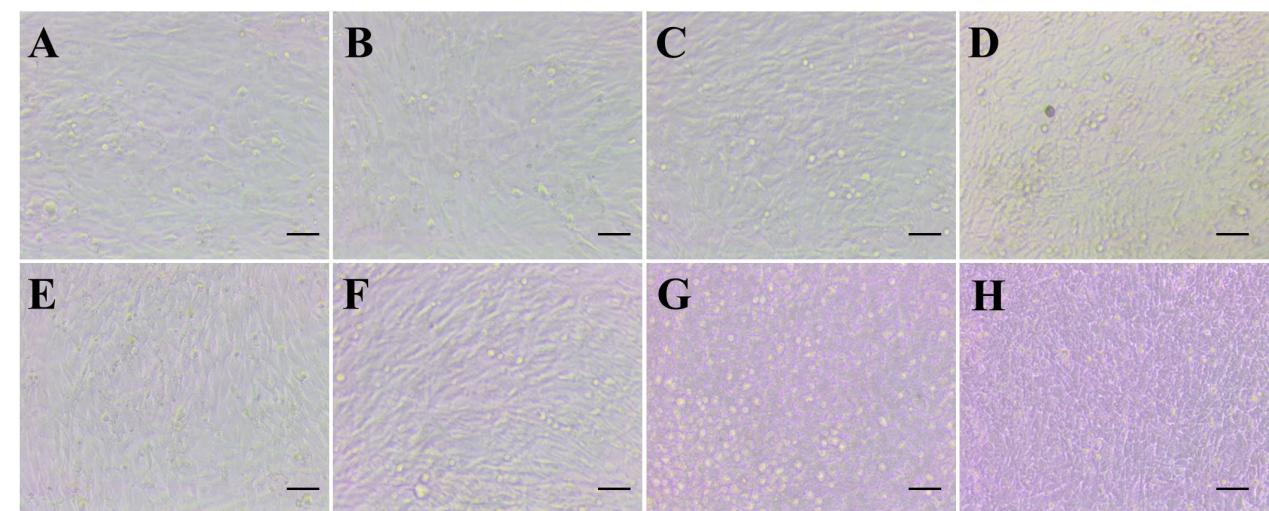


**Figure S9.** The effect of compound **2** on the morphology of UCB MSC cells at concentrations of 256 (A), 128 (B), 64 (C), 32 (D), 16 (E), and 8 (F) μg/mL, viewed under an eyepiece (10×) and an objective lens (20×), following co-culture for 48 h. Cell morphology of the negative control (G) and the blank control (H). Scale bar = 100 *μ*m.


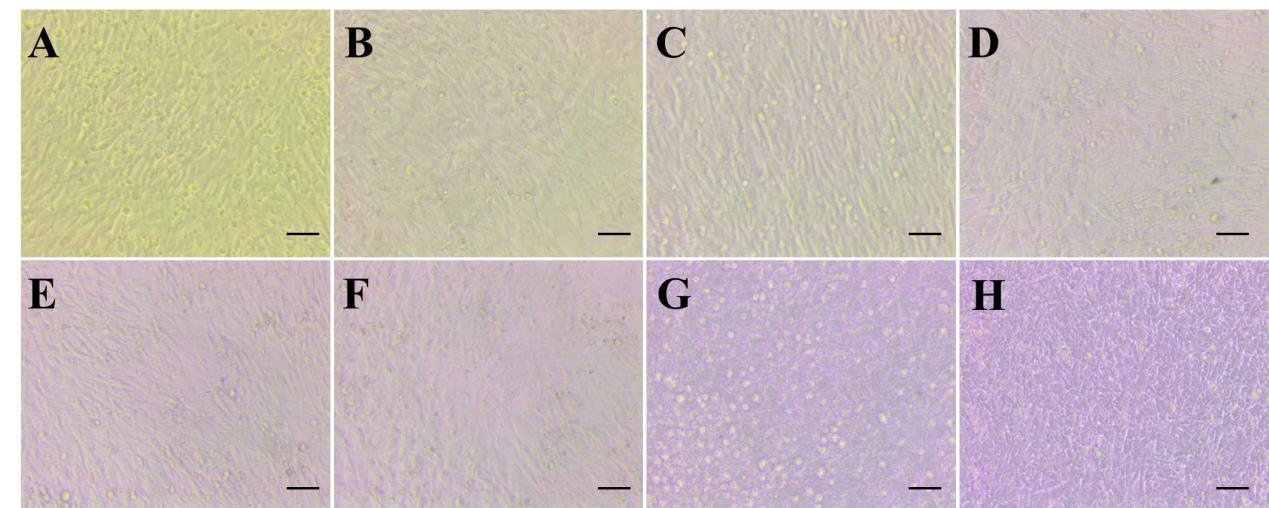


**Figure S10.** The effect of compound **4** on the morphology of UCB MSC cells at concentrations of 256 (A), 128 (B), 64 (C), 32 (D), 16 (E), and 8 (F) *μ*g/mL, viewed under an eyepiece (10×) and an objective lens (20×), following co-cultured for 48 h. Cell morphology of the negative control (G) and the blank control (H). Scale bar = 100 *μ*m.
